# Editorial Note: Enhanced audience sentiment analysis in IoT-integrated metaverse media communication

**DOI:** 10.1371/journal.pone.0354931

**Published:** 2026-07-30

**Authors:** The PLOS One Editors

The *PLOS One* Editors issue this Editorial Note to inform readers of the following issues that were noted after this article’s [1] publication:

- The article cited as Reference 16 was retracted while [1] was under review.
- The articles cited as References 1, 2, 3, and 4 do not appear to support the corresponding cited statements.
- The Editors have concerns about compliance with the PLOS policy on Artificial Intelligence Tools and Technologies.

Corresponding author JH stated that references 1–4 provide baseline methodological context. The Editors remain concerned that these references are not relevant as the cited context is not related to methodology.

The author stated that no generative AI tools were used for the generation of text in the article, but acknowledged that non-generative tools were used for text translation and polishing.
